# COVID-19 attributed Changes of Home and Family Responsibilities among Single Mothers

**DOI:** 10.1177/0192513X221105247

**Published:** 2022-05-27

**Authors:** Lisbeth A. Pino Gavidia, Hoda Seens, James Fraser, Marudan Sivagurunathan, Joy C. MacDermid, Laura Brunton, Samantha Doralp

**Affiliations:** 1 Department of Health and Rehabilitation Sciences, 6221 Western University, London, ON, Canada; 2 432162 Windsor University School of Medicine, Cayon, ON, Canada; 3 Department of Computer Science, University of Guelph, Guelph, ON, Canada; 4 Women's College Research Institute, 574481 Women's College Hospital, Toronto, ON Canada.; 5 Roth McFarlane Hand and Upper Limb Centre, St. Joseph’s Health Care London, London, ON Canada

**Keywords:** COVID-19, family responsibilities, home and family work role, unpaid work, single mothers

## Abstract

Lockdown measures during COVID-19 have presented increased challenges in the home and family responsibilities. Single mothers may face unique challenges as they may be isolated from external family supports. Changes on a 19-item home and family work role survey pre- and post-COVID were tested with a paired t-test and sign test; the impact of age and people in the home was assessed using linear regression. There was a significant increase (p < 0.05) in 6/19 post-COVID-19 family responsibilities. In comparison to pre-COVID-19, post-COVID-19 single mothers reported a statistically significant median increase in family responsibilities (Mdn = .0000), p < .041. Each additional person living in the home was associated with a decrease in family responsibilities (B = -13.1, 95% CI [-21.943, -4.247]). Changes in the home and family responsibilities confirm that COVID-19 led to increased unpaid work to fulfill home and family responsibilities among single mothers.

The coronavirus disease (COVID-19) caused by SARS-CoV-2 virus has quickly impacted every sphere of society. Emergent research regarding COVID-19 revealed that stay-at-home orders resulted in increased family responsibilities (Craig, 2020). Sarker (2021) demonstrated that family responsibilities have increased by 58% during quarantine measures. The impact of the unprecedented lockdown has exacerbated families’ care needs (Bahn et al., 2020). According to Craig and Churchill (2020), “care is often multitasked, and its magnitude is underestimated if a simultaneous activity is overlooked” (p. 6). The increased burden of care is the work devoted to the well-being of others, in particular focused on cleaning and cooking (Fortier, 2020). During the COVID-19 pandemic, there were observed increases of 38% and 25% in cleaning and cooking responsibilities, respectively, tasks primarily performed by women. Both of these family responsibilities are considered the most time-consuming chores necessary for the family’s survival and are classified as unpaid labor (Fortier, 2020).

The challenges of adhering to COVID-19–related protocols exposed vulnerabilities of women, in particular single mothers. For example, women are more likely to be single parents and already face gaps in family responsibilities prior to COVID-19 (Bahn et al., 2020; Choi et al., 2020). The COVID-19 pandemic amplified the importance of accounting for unpaid labor among single mothers as they are responsible for the entire family’s care. Before COVID-19, women were engaged in far more household tasks than men globally (Fortier, 2020). The International Labour Organisation (ILO) estimated that women and girls, worldwide, spend 4 hours and 25 minutes on average per day performing family responsibilities that correspond to 75% of all household tasks (Power, 2020). Similarly, during the pandemic, Chauhan (2020) demonstrated that the increased burden of family responsibilities fell disproportionally on mothers, making them more vulnerable than the rest of the population.

As priorities and responsibilities shifted during COVID-19, single mothers were responsible for keeping their families functioning in terms of household tasks, childcare, and home-schooling. For full-time employed single mothers, COVID-19–related closures of childcare centers and school presented a real challenge and many mothers struggled during this time (Collins et al., 2021; Yerkes et al., 2020). The lack of childcare and schooling forced mothers to reduce working hours and even abandon the labor market (Shafer, Scheibling, & Milkie, 2020). Single mothers experienced a dramatic increase in family responsibilities, leaving insufficient time for employment. As the care needs of the family increased during COVID-19, “single mothers felt tired, stressed and guilty because they were unable to compartmentalize paid work and family” (Hertz, Mattes, & Shook, 2020, p.23). The disruption associated with COVID-19 reinforced the need for recognition to mothers’ performance within the home; such as household tasks, added childcare, and schooling (Bahn et al., 2020; Power, 2020). The burden of childcare and home-schooling was incorporated as household tasks (Hupkau & Petrongolo, 2020). However, single mothers living in multi-adult households with shared responsibilities were able to manage better than those living in single-adult homes (Hertz et al., 2020).

As businesses closed or scaled back, the job market shrunk and this created major impediments in terms of available jobs. Single mothers were more likely to experience job loss and lay-offs (King et al., 2020). The public recommendations to stay-at-home directly exposed single mothers to a rapid reconfiguration of home and family responsibilities (Shafer et al., 2020). Mothers prioritized the essential service of caring for their families as nurturing and self-sacrificing day-to-day, especially through these unprecedented challenges for being accountable to their home and family (Craig & Powell, 2013). However, as single mothers were confined within the home, family responsibilities turned into pandemic exhaustion, requiring their intensive engagement in unpaid labor during the shutdown. The home became the facility for daily care routines with the primary responsibility in caretaking.

Understanding changes in family responsibilities could reveal the unique impact of COVID-19 on vulnerable families headed by women. There is evidence that the global pandemic impacted home and family responsibilities, and that single mothers have been especially impacted by societal changes due to COVID-19. However, most studies have focused on specific aspects of family responsibilities like child care and not on a full range of common household tasks and roles. More research is needed to understand the nature of task role changes and the factors that are associated with changes in workload related to family responsibilities. The primary purpose of this study was to identify changes to workload in family responsibilities before and after COVID-19. In addition, a secondary purpose of this study was to examine the relationship between a single mother’s age and number of people living in the home to determine the variation in family responsibilities post-COVID-19.

## Methods

### Data Source

The Home and Family Work Role Survey was administered online from June 26 to August 31, 2020 and therefore represents the pre-vaccination phase of the pandemic (MacDermid, 2018). The online survey, of which “The Home and family Work Role Survey” was a subset, asked participants closed-ended questions about demographic information and changes in family responsibilities, substance use, anxiety, and depression before and after the pandemic. Respondents were asked to fill out the survey once and reported retrospectively pre-COVID-19 (before March 11, 2020) and post-COVID-19 (after March 11, 2020). This study data was extracted from the larger survey that recruited in a broad sample, to focus only on those respondents who self-reported as being single mothers (*n* = 72).

### Measurement of Family Responsibilities

The Home and Family Work Role Survey consisted of 19 items (Table 1). Participants rated from 0 to 100-point scale, indicating what percentage of each item was usually done by them, not by family, friends, or paid staff. If the item was not relevant to respondents, then the option was “not applicable” to them. Although the item “help children with homework” was part of the Home and Family Work Role Survey pre-pandemic, an additional related item was incorporated “supervise children with homework” given the importance of home-schooling during the pandemic and the new hands-off/on-call responsibility associated with supervising home-schooling (Craig & Churchill, 2021). The instrument was designed purposely to include gendered items that are typically performed by women, typically performed by men, or less gendered items to explore the gendered nature of family responsibilities to avoid gender bias.

**Table 1.** COVID-19 Individual Items of Home and Family Work Role Amongst Single Mothers.

Home and family work role (19/100)	Pre-COVID-19		Post-COVID-19 Post–Pre				Potential explanatory factors
	Mean	SD	Mean	SD	t	Sig.	
1. House cleaning (*n* = 63)	6.5	2.9	7.1	2.9	2.0	.045*	Increased need for disinfecting procedures; decreased external supports; increased time at home
2. Outdoor maintenance (*n* = 52)	5.0	3.7	6.0	3.4	3.9	.000**	Increase garbage due to greater time at home and disinfecting procedures; decreased external supports
3. Laundry (*n* = 63)	7.1	3.1	7.3	3.1	.8	.434	No change
4. Home decorating (*n* = 40)	6.0	4.3	6.5	4.1	2.3	.024*	Increased opportunity to do decorating work at home as more time at home; decreased external supports
5. Home repairs (*n* = 40)	4.7	4.0	5.6	3.9	2.6	.014*	As #4
6. Mowing lawn/shoveling snow (*n* = 32)	4.0	4.4	4.1	4.3	.7	.475	No change; not a major concern given the pandemic and the time of year
7. Garden (*n* = 44)	5.5	4.2	5.8	4.1	1.8	.084	No change; not a major concern given the pandemic and the time of year
8. Prepare meals (*n* = 63)	6.7	3.2	7.1	3.1	1.5	.140	No change; persistent high level of responsibility
9. Shop for groceries and supplies (*n* = 62)	6.9	3.3	7.5	3.1	2.3	.024*	More meals at home; more need for pandemic-related supplies; “stocking up” behaviors, more person hours of people in the home, less external supports
10. Drive family to appointments and activities (*n* = 52)	7.2	3.4	7.4	3.5	.4	.662	No change as reduced activities, persistent high level of responsibility
11. Arrange family appointments (*n* = 51)	7.5	3.6	7.5	3.7	−.090	.928	No change; persistent high level of responsibility
12. Maintain vehicles (*n* = 50)	6.1	4.0	5.9	3.8	−.693	.492	No change
13. Help children with homework (*n* = 42)	6.9	3.8	7.1	3.7	.755	.455	No change; as primary parent already carrying most responsibility for helping children with homework; online learning was not fully operationalized at the stage of the pandemic
14. Supervise children with homework (*n* = 36)	6.7	3.8	7.2	3.9	.292	.205	As #13
15. Care for children in the home (*n* = 47)	7.1	3.2	8.1	2.8	2.8	.008*	Increase time of children being in the home; less daycare and other external supports
16. Care for children when sick (*n* = 43)	7.9	3.6	8.2	3.3	.9	.398	No change as rate of illness for children was not dramatically affected by the pandemic; persistent high level of responsibility
17. Care for other family members (*n* = 27)	5.1	3.8	5.6	3.8	1.3	.206	No change as caregiving focused on children as above
18. Earn family income (*n* = 49)	6.9	3.9	6.5	4.3	–1.700	.096	Small insignificant decrease which may be related to job loss or underemployment
19. Manage family finances (*n* = 49)	7.8	3.8	7.7	3.8	−.545	.588	No change persistent high level of responsibility

*Note.* Two-tailed independent samples T-test was used for group comparisons pre- and post-COVID-19. Source: Home and Family Work Role Survey, 2020. Each item is scored out of 10, so scores greater than 5 indicate that women carry more than half of the workload.

*p < 0.05. **p < 0.005

### Data Analysis

The primary research question: “what is the impact on family responsibilities because of the changes from COVID-19?” was analyzed in four ways. First, the mean percentage scores in each item were presented for the proportion of workload performed pre- and post-COVID-19. Second, the change scores (post**–**pre-COVID-19) were compared through paired t-tests to identify the significance of individual changes. Third, the sign test was considered to report the overall change as an alternative to the t-test because the distribution of differences between paired observations was not normal. In addition, seven extreme outliers were detected that were more than 3 box-lengths from the edge of the boxplot. Therefore, we used a nonparametric test rather than the t-test for the overall change score. Finally, multiple linear regression was used to examine the magnitude and precision in predicting the sum of the total family responsibilities post-COVID-19 based on two predictor variables: mothers’ age and people living at home.

## Results

There were 72 single mothers (41 single, 30 divorced, and one widowed) from the original sample of 1847 participants. Pre**–**post pandemic family role responsibility individual items means and standard deviations and potential reasons for changes observed are listed in Table 1. Pre-COVID-19 mean percentage scores were higher in 4/19 items: manage family finances/bills (70.8%), arrange family appointments and activities (70.5%), earn family income (60.9%), and maintain vehicles (60.1%). Post-COVID-19 higher mean percentage scores were found in 15/19 items but not statistically different in 13/19: care for children when sick (80.2%), care for children in the home (80.1%), shop for groceries and supplies (70.5%), drive family to appointments and activities (70.4%), laundry (70.3%), supervise children with homework (70.2%), help children with homework (70.1%), prepare meals (70.1%), house cleaning (70.1%), home decorating (60.5%), outdoor maintenance (60%), garden (50.8%), care for other family members (50.6%), home repairs (50.6%), and mowing lawn/shoveling snow (40.1%). Care for children in the home showed the highest burden of family responsibilities during lockdown measures to curb COVID-19. Six of these items showed statistically significant changes. These included the following: (1) Care for children in the home *t* (46) = 2.771, *p* > .05.2) Shop for groceries and supplies *t* (61) = 2.308, *p* < .05.3) House cleaning *t* (62) = 2.048, *p* < 0.05.4) Home decorating *t* (39) = 2.348, *p* < .05.5) Outdoor cleaning *t* (51) = 3.860, *p* < .001.6) Home repairs *t* (39) = 2.559, *p* < .05.

Table 2 reports the overall score change based on a binomial distribution of the sign test. In this section, we described our findings for the overall change of family responsibilities post-COVID-19. Seventy-two single mothers contributed scores to assess the overall change of family responsibilities measured pre- and post-COVID-19. An exact sign test compared the median of the paired differences, which indicated a statistically significant increase in family responsibilities post-COVID-19 (Mdn = .0000), z = 2.04, *p* < .041.

**Table 2.** COVID-19 Overall Score Change of Home and Family Work Role Amongst Single Mothers (*n* = 72).

	Related-Samples Sign Test Summary
The median of differences between family responsibilities pre- and post-COVID-19	.0000
Standardized test statistic	2.041
Exact sig. (2-sided test)	.041*

Source Home and Family Work Role Survey, 2020.

*p < 0.05.

Table 3 presents the linear regression model that indicated statistically significant changes in family responsibilities post-COVID-19*, F* (2, 69)= 18.540, *p* < .001. Both a single mother’s age and number of people living in the home contributed significantly to the prediction of family responsibilities. Results from linear regression analysis (Table 2) revealed a significant positive beta coefficient for mother’s age (B = 1.648, *p* < .05) and a significant negative beta coefficient for people living in the home (B = −13.095, *p* <.05). The overall model fit had an R^2^ of 35% with an adjusted R^2^ of 33%, a small size effect according to Cohen rules. The adjusted R^2^ explains that the inclusion of all the predictor variables into this regression model explained 33% of the variability of family responsibilities post-COVID-19 over and above the mean model.

## Discussion

This study confirms that the COVID-19 pandemic was associated with an increase in family responsibilities for single mothers, particularly in gendered roles like meals and cleaning tasks. Before COVID-19, family responsibilities fell disproportionally onto women’s shoulders (Sarker, 2021). This was confirmed in this study since women performed more than half of listed tasks (15/19) pre-pandemic. Since the survey was designed to capture typically gendered tasks done by both men and women, this indicates that even prior to the pandemic our sample was performing the majority of family role tasks. This is consistent with findings across a number of epidemiological and qualitative studies. For instance, in a commentary in The Lancet more than 75% of the housework was done by women, that corresponded to 9% of the gross domestic product globally (King et al., 2020). In a quantitative study, single mothers reported that performing domestic chores is time-consuming: “Yet there are only 24 hours in the day, and if time demands conflict too much, something must give. Often this is [our] wellbeing” (Craig, 2020, p.3). A policy brief found that most of the family responsibilities that women performed at home were highly gendered, including multitasking (Power, 2020). Labors referred to as multitasking were carried out simultaneously or by switching from one to another (Powell & Craig, 2015). This is consistent with the findings in our study that the 3 tasks where women did not do the majority of the work pre-pandemic were tasks considered more typically gendered for men, for example, outdoor maintenance, home repairs, and mowing lawn/shoveling snow. Further, the tasks where women performed the highest workloads were typically gendered for women, for example, arranging appointments, laundry, and preparing meals. During COVID-19, the escalation of intensive mothering was centered around household tasks, childcare, and home-schooling (Collins et al., 2021). This study reveals that caring for children in the home was the highest burden of family responsibilities, with the potential explanation being the closure of childcare and schools due to lockdown measures. This trend was consistent with prior research that increased childcare responsibilities fall onto women during the pandemic (Hupkau & Petrongolo, 2020).

Prior to COVID-19 single mothers already struggled with an overload of family responsibilities, but the pandemic resulted in intensification of their unpaid labor. The survey was designed to evaluate the distribution of work within families. Table 1 indicates the variable changes in work roles during the pandemic and potential explanations. We identified significant changes in 6/19 family responsibilities post-COVID-19: (1) care for children in the home, (2) shop for groceries and supplies, (3) house cleaning, (4) home decorating, (5) outdoor maintenance, and (6) home. This emphasizes why it is important to evaluate family responsibilities at a task level since some activities increased, others decreased, and some remained the same. Most of the activities that changed are consistent with the nature of public health polices during the pandemic. When the lockdown started in March 2020, care for children in the home increased as daycares and others external supports were closed. The need to shop for groceries and supplies increased as more meals were provided at home, and more personal care and cleaning were performed within the home. House cleaning increased due to greater time at home and the need for disinfecting procedures. The fact that home decorating increases may be more of an indirect effect of the pandemic, since the opportunity to do work within the home was increased and people may have felt this was a time to do some these less urgent home tasks. Since construction was deemed an essential service, related stores and services remained open and institute pickup services meaning that people could safely access supplies and work on home projects. Since the survey was administered and the summer season was approaching this might have influenced outdoor activities.

**Table 3.** Linear Regression Estimates Predicting Family Responsibilities Post-COVID-19 for the Overall Sample (*n* = 72).

Predictor	Adjusted beta coefficient (95% CI)	Test statistic	*p*-Value	Explanation
F-test (F (ndf, ddf), *p*-value)		18.5 (2, 69)	<0.001	
Mother’s age	1.6 (.592, 2.705)	3.1	<0.05	Each year added to mother’s age increases percentage workload by 1.6/72
People living at home	−13.1 (-21.943, - 4.247)	−2.9	<0.05	Each additional person in the household reduces total workload by 13.1/72

R^2^= 0.35; ∆ R^2^ = 0.33

Thirteen family responsibilities did not show statistically significant post-COVID-19: (1) supervising children with homework, (2) care for other family members, (3) earn family income, (4) prepare meals, (5) garden, (6) help children with homework, (7) care for children when sick, (8) laundry, (9) maintain vehicles, (10) mowing lawn/shoveling snow, (11) drive family to appointments and activities, (12) manage family finances/bills, and (13) arrange family appointments and activities. While overall these tasks were areas less impacted by the pandemic, high standard deviations also indicate that these tasks were highly variable between families. An important consideration is the survey asked mothers to report the proportion of the household tasks performed rather than the volume of the workload. The volume of workload can increase but the amount/proportion a person does could remain the same. For example, at the extremes if a single mother is doing 100% of a given item and the workload increases, there will still be no change observed if the mother is doing 100% of the task. Similarly, if mothers had reduced hours/income related to work outside the home, they could still be doing the same proportion of that role within the family. Since the survey was designed to look at workload distribution, not amount of work performed some nuanced changes may not have been revealed on the survey. Therefore, we expect that our data indicates both increased family responsibilities and decreased supports from others in completing that work

Our regression model indicated that an increase in mother’s age of 1 year is associated with an increase of one-unit change in family responsibilities post-COVID-19. The reasons why older mothers perform a greater percentage workload are not clear but may relate to having older parents who are less able to provide instrumental help, the age of children, or that older mothers were less likely to have another adult in the home. Qualitative analyses or more detailed information on family structure would be needed to investigate these hypotheses. In contrast, an increase in number of people living in the home was associated with a decrease in individual family responsibilities post-COVID-19. Possible reasons for the decrease include that other adults in the household take on a percentage of the workload, for example, extended family; or that older children take on substantive roles in performing household tasks. Our findings are consistent with studies suggesting that women from multi-adult households experienced fewer challenging situations than women who live alone with their children (Hertz et al., 2020). The consistently higher beta weights indicated that the associations in the number of people living in the home have a greater influence than the mother’s age.

Others have suggested that stay-at-home regulations have increased family responsibilities for women (Craig & Churchill, 2020). Women are more likely to be the sole providers than men (Hupkau & Petrongolo, 2020), so single parenthood is a factor that compounds family role responsibilities for women to a greater extent than men. Mothers sacrifice their well-being to meet the needs of their children, particularly when the mother is the primary caregiver (Russell et al., 2020). This study revealed that single mothers already assumed more than half of the workload in many aspects of family role responsibilities prior to the pandemic, but that shutdowns exacerbated their disproportionate family responsibilities. The interrelated challenges due to loss of childcare, schooling, and jobs exacerbated family responsibilities (Bice-Wigington et al., 2015). The long-term consequences of these disproportionate challenges are not yet known for single mothers. Future research should examine mothers’ mental health and well-being during the pandemic. In these unprecedented times, it would be helpful to have greater understanding of the impact of societal changes on vulnerable groups, such as single mothers to influence restructuring of governing policy to provide greater protection.

### Limitations

The strengths of this study include a standardized questionnaire and focus on a specific subgroup. We acknowledged several limitations that should be noted. The sample size was not sufficient to examine subgroups of single mothers and we recognize that there is substantial diversity in family structures even within households led by a single parent. When we looked at the individual items of home and family work roles, the number of respondents varies because some of the items were not relevant to single mothers. We used a previously validated survey which asks respondents to rate the percentage of workload rather than the amount of workload performed. While this is an important perspective it can miss situations where the amount of work changes, but the proportion does not. Since we expected that loss of external help may be a major issue for single mothers during the pandemic, we felt that this validated measure best captured the construct we were interested in. We did not have data on the ages of the children or adults in the home and could not consider many predictors based on our sample size, to avoid overfitting the model. We were unable to consider employment within the model since there were too many small cells that could not be meaningfully recombined. Additionally, the full range of family responsibilities is not yet well-established because of the multiple roles single mothers undertook during the pandemic (Hertz et al., 2020), so this survey reflects only a sampling of the work performed.

## Conclusion

We found that single mothers performed more than half of the family responsibilities (15/19) pre-pandemic and that increases in 6/19 tasks/roles including: care for children in the home, shop for groceries and supplies, house cleaning, home decorating, outdoor maintenance, and home repairs. Having more people living in the home to share family responsibilities during the COVID-19 pandemic may have lessened the impact on the single mother. Single mothers may have lost external supports during the pandemic and, as they resume more normal work and occupation, their needs to rebuild support systems should be factored into their return to work and activities. Further studies should focus on the impact on mothers’ mental health and their ability to create a healthy family environment based on different family structural features, that is, whether single mothers live by themselves, with their children or in multi-adult households.
